# The Identification of Key Gene Expression Signature and Biological Pathways in Metastatic Renal Cell Carcinoma

**DOI:** 10.7150/jca.38379

**Published:** 2020-01-16

**Authors:** Lin Bao, Ye Zhao, ChenChen Liu, Qi Cao, Yu Huang, Ke Chen, Zhengshuai Song

**Affiliations:** 1 Department of Urology, Union Hospital, Tongji Medical College, Huazhong University of Science and Technology, Wuhan 430022, China.; 2 Department of Urology, The Central Hospital of Wuhan, Tongji Medical College, Huazhong University of Science and Technology.; 3 Cancer Center, Union Hospital, Tongji Medical College, Huazhong University of Science and Technology, Wuhan 430022, China.

**Keywords:** clear cell renal cell carcinoma, metastasis, biomarker, diagnosis, prognosis.

## Abstract

**Purpose:** To investigate the potential mechanisms contributing to metastasis of clear cell renal cell carcinoma (ccRCC), screen the hub genes, associated pathways of metastatic ccRCC and identify potential biomarkers.

**Methods:** The ccRCC metastasis gene expression profile GSE47352 was employed to analyze the differentially expressed genes (DEGs). DAVID was performed to assess Gene ontology (GO) and the Kyoto Encyclopedia of Genes and Genomes (KEGG) analyses. The protein-protein interaction (PPI) network and modules were constructed. The function pathway, prognostic and diagnostic analysis of these hub genes was picked out to estimate their potential effects on metastasis of ccRCC.

**Results:** A total of 873 DEGs were identified (503 upregulated genes and 370 downregulated genes). Meanwhile, top 20 hub genes were displayed. GO analysis showed that the top 20 hub genes were enriched in regulation of phosphatidylinositol 3-kinase signaling, positive regulation of DNA replication, protein autophosphorylation, protein tyrosine kinase activity, etc. KEGG analysis indicated these hub genes were enriched in the Ras signaling pathway, PI3K-Akt signaling pathway, HIF-1 signaling pathway, Pathways in cancer, etc. The GO and KEGG enrichment analyses for the hub genes disclosed important biological features of metastatic ccRCC. PPI network showed the interaction of top 20 hub genes. Gene Set Enrichment Analysis (GSEA) revealed that some of the hub genes was associated with metastasis, epithelial mesenchymal transition (EMT), hypoxia cancer and adipogenesis of ccRCC. Some top hub genes were distinctive and new discoveries compared with that of the existing associated researches.

**Conclusions:** Our analysis uncovered that changes in signal pathways such as Ras signaling pathway, PI3K-Akt signaling pathway, etc. may be the main signatures of metastatic ccRCC. We identified several candidate biomarkers related with overall survival (OS) and disease-free survival (DFS) of ccRCC patients. Accordingly, they might be novel therapeutic targets and used as potential biomarkers for diagnosis, prognosis of ccRCC.

## Introduction

Renal cell carcinoma (RCC) is one of the most frequently malignant tumors in the urinary system. Renal cell carcinomas (RCC) contribute to an evaluated 338,000 new cancer diagnoses and 144,000 tumors related deaths in 2012^1^. Although the pathological types of RCC are diverse, the clear cell renal cell carcinoma is the most common type 2. Although the treatment of renal cancer had achieved great results, many patients with advanced stage, especially who with metastatic tumors, still have poor prognosis. Metastasis is the leading cause of cancer death 3. In this respect, intervention at the point of metastasis will be more valuable than at time when ccRCC has advanced to later stages. Thus, it is highly desirable to effectively estimate ccRCC with increased metastasis risk. Identifying effective biomarkers to better predict the diagnostic and prognostic levels of this malignancy is also of vital importance.

The current exploitation of high-throughput gene microarray to analyze normal and tumor tissue samples from patients confers us an opportunity to detect and explore the comprehensive molecular landscapes of tumors at multiple levels ranging from somatic mutations and copy number alteration at the genome level to gene expression changes at transcriptome level 4-6. While, the use of microarrays in clinic is greatly restricted because of countless genes detected by gene profiling, lack of independent stability, likewise the complex statistical analyses. Meanwhile experimentally identifying key genes in genome-wide is a waste of time and formidable. To apply these expression profiles in clinical practice as soon as possible, it is necessary to develop an optimal method that could be handled by routine detection. Moreover, there is a clear need to improve our ability to discovery ccRCC patients with high risk of metastasis. The challenge of accurately predicting ccRCC metastasis may be partly attributable to an intricate network of pathways that facilitate the disease development.

In present study, we downloaded GSE47352 from the Gene Expression Omnibus (GEO, http://www.ncbi.nlm.nih.gov/ geo/) and perform the GEO2R online tool to systematically measured the differentially expressed genes (DEGs). Then, we established protein-protein interaction (PPI) network of the DEGs and selected the top 20 hub genes by a high degree of connectivity. Furthermore, the GO and KEGG pathways of the 20 hub genes were explored. Meanwhile, overall survival (OS) and disease-free survival (DFS) analysis of the 20 hub genes were operated based on GEPIA (Gene Expression Profiling Interactive Analysis) database. Some of them not only associated with OS but also DFS. Receiver operating characteristic (ROC) curve analysis of the hub genes with both OS and DFS significance was performed. Several genes could adequately distinguish ccRCC from paired normal tissues with an area under the curve (AUC) of 0.9235-0.9451. After that, genes were selected to further evaluate the mRNA expression in normal and tumor sample tissues by TCGA. Finally, we focused our attention on AURKB, a member of the Aurora kinase subfamily that encoded a serine/threonine kinase, and regulated the arrangement and segregation of chromosomes during mitosis and meiosis by correlating with microtubules. Although some of previous studies displayed that AURKB may play a key role in the tumorigenesis and progression of several types of cancer^7^-^10^, the study of this gene in ccRCC had not been elucidated. To acquire further insight into the function of AURKB, we performed GSEA to map into GO analysis and KEGG pathways database. We also found that the expression of AURKB associated with clinical and pathological characteristics of patients with ccRCC and its expression levels were independent prognostic factors for ccRCC. In conclusion, our study identified 20 hub genes, which may play leading roles in ccRCC progression. Some dispensable biological function pathway in metastatic ccRCC was identified. This study also demonstrated that AURKB may be a novel biomarker for predicting the diagnosis and prognosis, and may be an important target for the treatment of metastatic ccRCC.

## Materials and methods

### Microarray Data

Gene expression profile of GSE47352 was download from the GEO database, which was a free and open available database. The GSE47352 dataset has a total of 9 samples, containing 5 primary ccRCC samples, 4 metastasis ccRCC tissues, according to agilent GPL570 platform (Affymetrix Human Genome U133 Plus 2.0 Array). It includes genome-wide mRNA expression data of this 9 samples.

### Identified genes of differential expression

The DEGs between metastasis and non-metastasis ccRCC samples were analyzed using GEO2R https://www.ncbi.nlm.nih.gov/geo/geo2r/, based on R language, which was an online analysis tool for the GEO database. We defined DEGs as differentially expressed with logFC > 2 (upregulated genes) or logFC < - 2 (downregulated genes), according to the criteria 11, 12. The *P* value < 0.05 was considered statistically significant, which was utilized to decrease the false positive rate. Then, 503 upregulated genes and 370 downregulated genes were found, and the top 20 genes with a high degree of connectivity were chose as hub genes.

### Gene Ontology and KEGG Pathway Analysis of DEGs

Genes could be annotated by Gene ontology (GO) analysis and their functions were classified by biological pathways, molecular function, as well as cellular components 13. The Kyoto Encyclopedia of Genes and Genomes (KEGG) is a set of databases that could dispose biological pathways and genomes related to diseases and drugs. KEGG substantially is a channel for the overall and deep understanding of biological systems 14. The cut-off criterion with statistic difference was P < 0.05. Used the DAVID online database (DAVID, http://david.ncifcrf .gov), cellular components, molecular functions, biological processes, and pathways of DEGs were analyzed.

### PPI Network Analysis

The protein-protein interaction (PPI) information, like physical and functional associations, was assessed and integrated by The Search Tool for the Retrieval of Interacting Genes (STRING), an online tool. Until now, STRING version 10.0 has covered a total of 9,643,763 proteins from 2031 organisms 15. To estimate the interactional correlation of these DEGs, DEGs was first drawn by STRING and then the Cytoscape software was used to construct a PPI network and module. Also, STRING was used to map 20 hub genes according to maximum number of interactors ≤ 5 and confidence score ≥ 0.4. GO and KEGG pathway was also utilized to analysis their potential information.

### The Hub Gene Expression Level

In this study, the boxplot was employed to visualize the expression of 2 hub genes in 533 ccRCC samples and 72 normal renal samples from TCGA-KIRC (clear cell renal cell carcinoma) dataset. The Human Protein Atlas (HPA, https://www.proteinatlas.org/) is a Swedish-based project, which was launched in 2003 with the goal to map all human proteins in organs, tissues, cells and using the integration of diverse omics technologies 16. By acquiring immunohistochemical data of patients with or without ccRCC based on HPA, we further confirmed the expression of the two hub genes.

### Survival Analysis of Hub Genes

The overall and disease-free survival information was based on GEPIA database. The hazard ratios (HR) with 95% confidence intervals were calculated and *P* < 0.05 was regarded as statistically significant.

### Gene Set Enrichment Analysis (GSEA)

533 ccRCC samples from TCGA were classed into two groups (high versus low) based on the mRNA expression level of AURKB, and the median expression value was believed to be the cut-off point. To explore the potential mechanism of AURKB, GSEA (http:// software.broadinstitute.org/gsea/index.jsp) was operated between the two groups. We selected annotated gene sets c2.cp.kegg. v5.2.symbols.gmt as the reference gene sets. FDR < 0.05 and gene size ≥ 100 were considered as the cut-off criteria.

### Tissue samples and Cell culture

Tissue samples and Cell culture executed as previously described 17.

### Transient transfection assay

The siRNA targeting AURKB (siAURKB) and the siRNA negative control (si-NC) were chemosynthetic by GenePharma (Shanghai, China). According to the manufacturer's recommendations, AURKB and si-NC with a final concentration of 50 nM were transfected with Lipofectamine® 2000 (Invitrogen, USA).

### Cell migration and invasion assays

Migration and invasion assays were implemented as previously described 17.

### Statistical Analysis

The values of each group were shown as the mean ± SD. A difference of *P* < 0.05 was considered statistically significant. The statistical analysis software performed in this study was GraphPad Prism 6.0 (GraphPad Software, Inc., USA) and SPSS 22.0 (IBM SPSS, Chicago, IL). Unpaired t-test was used to evaluate the statistical difference. And Mann-Whitney test was used to analyze the difference of AURKB expression in the ccRCC subgroups. The receiver operator characteristic (ROC) curve was used to analyze diagnostic values of AURKB in different patients with ccRCC. The association between the AURKB expression level and the OS, DFS rate were computed by the Kaplan-Meier curve and log-rank test.

## Results

### Screening of DEGs

**Figure 1:**
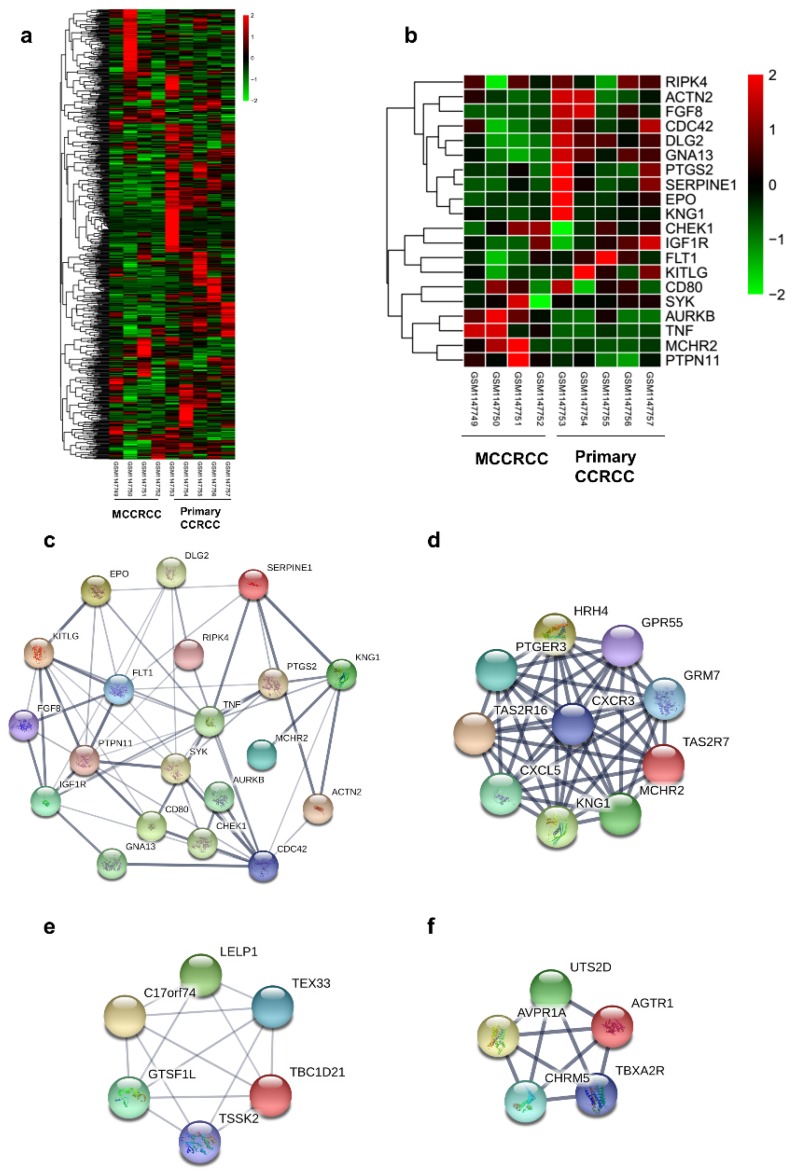
Protein-protein interaction network of the top 20 hub genes and modular analysis. (a)The DEGs of GSE47352. (b) The heatmap of 20 hub genes. (c)The PPI network of the top 20 hub genes. (d) module 1 (e) module 2 (f) module 3 of DEGs from PPI network.

There were 4 metastatic and 5 non-metastatic ccRCC samples in this study. The DEGs were identified by the GEO2R online analysis tool, using P value < 0.05 and |logFC| ≥ 2 as cut-off criteria. A total of 873 DEGs were detected after analyzing GSE47352, 503 of which were upregulated genes while 370 were downregulated (Figure 1a).

### Hub Genes Screening from the PPI Network

**Figure 2:**
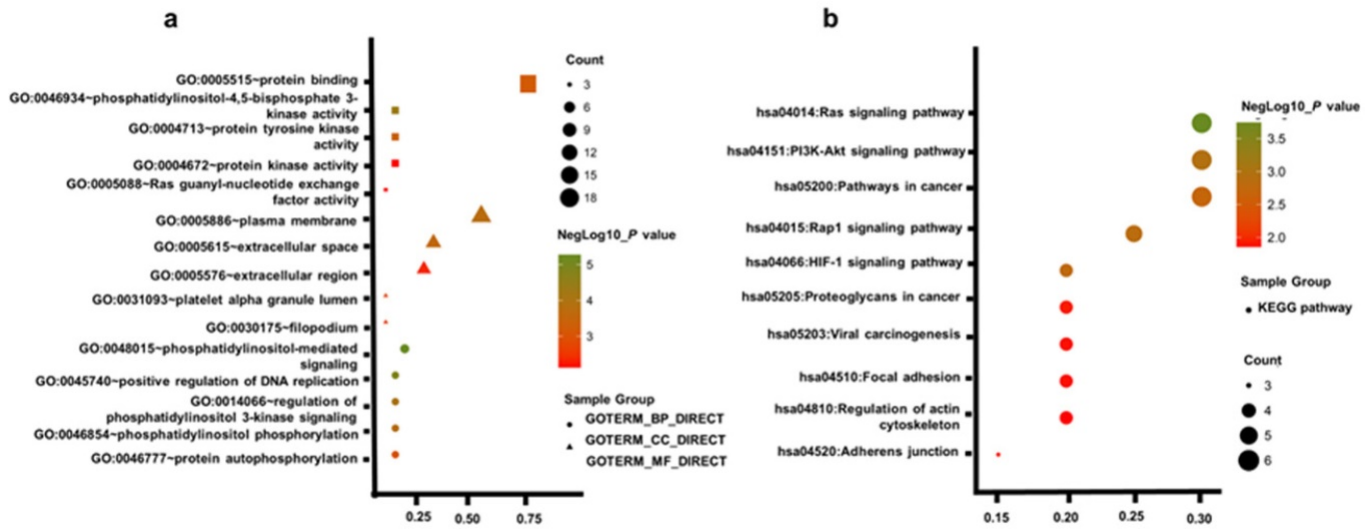
Gene Ontology enrichment analysis and KEGG pathways of top 20 hub genes (a) GO analysis of top 20 hub genes. (b) KEGG pathway of top 20 hub genes.

20 hub genes were identified, according to their degree of connectivity from high to low (Table 1) and the expression of them was presented by the heatmap (Figure 2b). According to the message of the STRING protein query, we built the PPI network of the top 20 hub genes based on the degree of connectivity (Figure 1c). The top 20 hub genes are as follows: RIPK4, TNF, CDC42, KNG1, PTPN11, KITLG, PTGS2, SYK, IGF1R, EPO, SERPINE1, FLT1, AURKB, GNA13, DLG2, ACTN2, CHEK1, FGF8, CD80 and MCHR2. Furthermore, MCODE plugin in Cytoscape was used to identify the top 3 significant modules from the PPI network (Figure 1d-f). Based on GO biological progress analysis, these modules were enriched in inflammatory response, G-protein coupled receptor signaling pathway, positive regulation of cytosolic calcium ion concentration and positive regulation of vasoconstriction (Table 4).

### Functional Enrichment Analysis

To acquire a more comprehensive and deep understanding of those chosen hub genes, DAVID was used to analysis GO function and KEGG pathway enrichment. The TOP5 gene ontology categories were shown in Table 1. In biological processes (BP), the hub genes were mainly enriched in phosphatidylinositol-mediated signaling; regulation of phosphatidylinositol 3-kinase signaling; positive regulation of DNA replication; phosphatidylinositol phosphorylation; protein autophosphorylation; And in molecular function (MF), these genes were mainly associated with phosphatidylinositol-4,5-bisphosphate 3-kinase activity, protein tyrosine kinase activity, protein binding, protein kinase activity, Ras guanyl-nucleotide exchange factor activity. In addition, GO cell component (CC) analysis shown that they were principally involving the plasma membrane, extracellular space, platelet alpha granule lumen, filopodium, and extracellular region (Table 2).

Table 3 uncovered the most significantly KEGG pathway of the top 20 hub genes. These genes were enriched in Ras signaling pathway, PI3K-Akt signaling pathway, Rap1 signaling pathway, HIF-1 signaling pathway, Pathways in cancer, Proteoglycans in cancer, Adherens junction, Viral carcinogenesis, Focal adhesion, Regulation of actin cytoskeleton. Figure. 2a, b gives a GO and KEGG pathway enrichment plot of these hub genes.

### The Kaplan-Meier survival analysis

**Figure 3:**
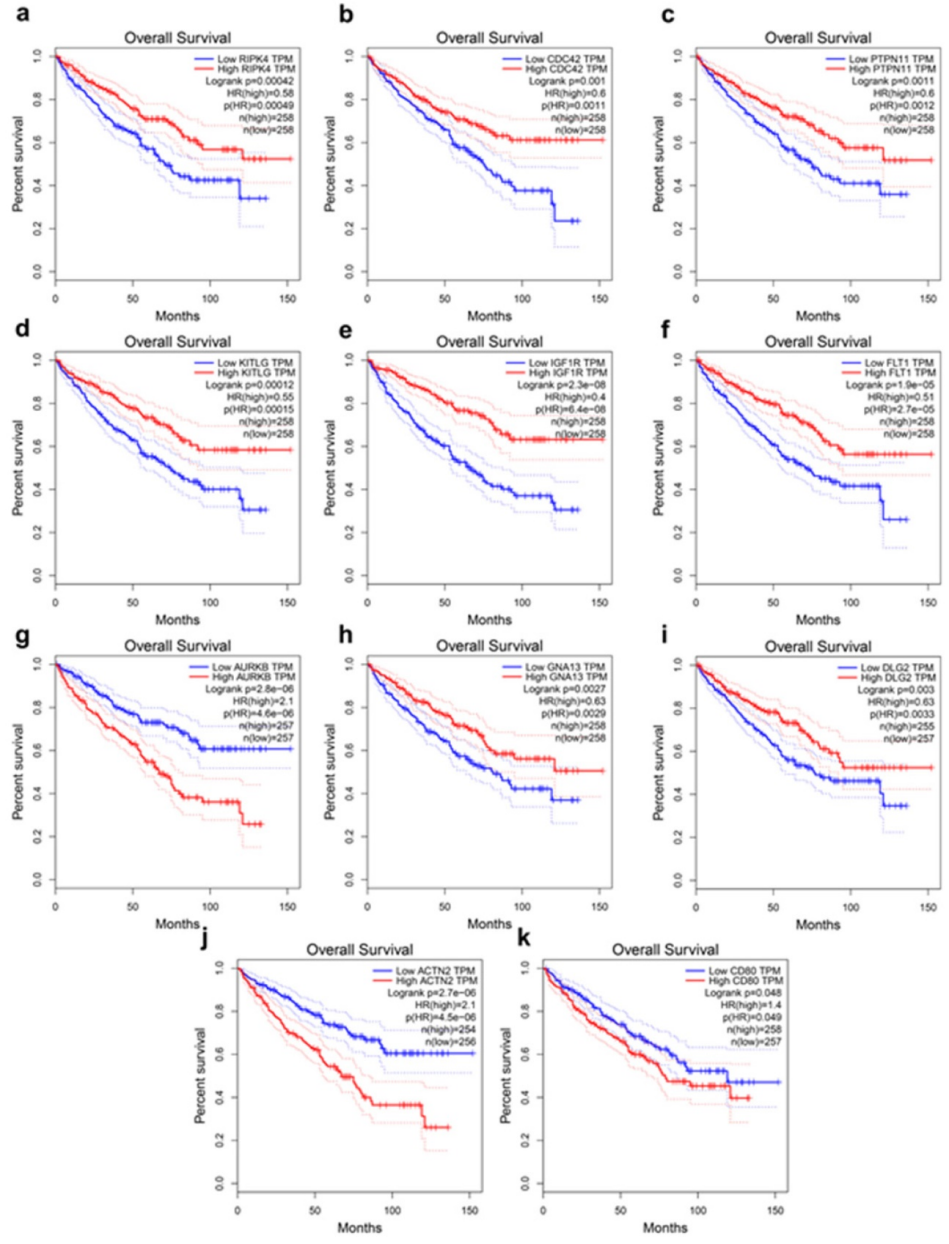
Hub genes have prognostic value of overall survival. (a) RIPK4 (b) CDC42, (c) PTPN11, (d) KITLG, (e) IGF1R, (f) FLT1, (g) AURKB, (h) GNA13, (i) DLG2, (j) ACTN2, (k) CD80. The overall survival information was based on GEPIA database, P < 0.05 was considered statistically different.

**Figure 4:**
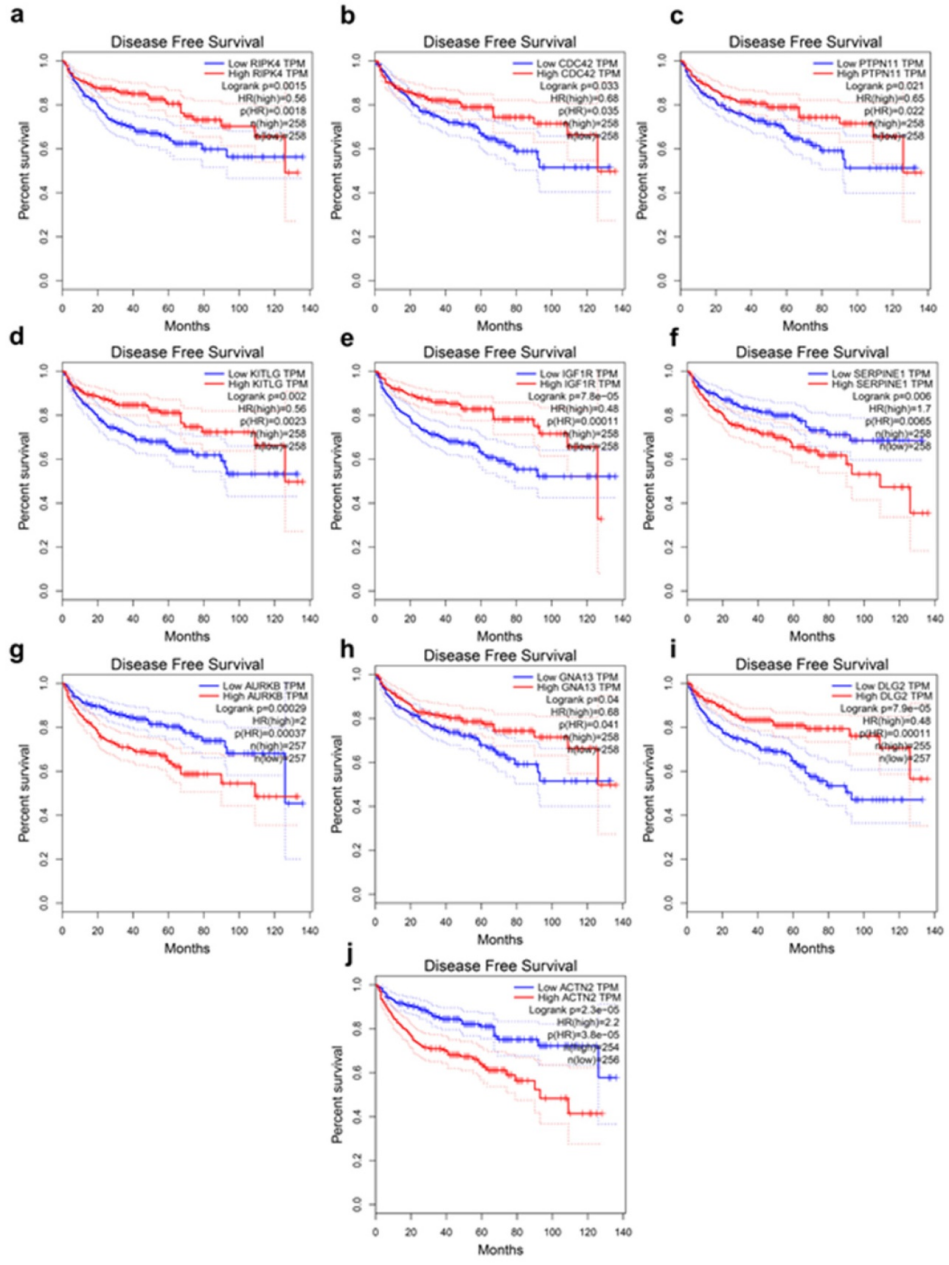
Hub genes have prognostic value of disease-free survival. (a) RIPK4, (b) CDC42, (c) PTPN11, (d) KITLG, (e) IGF1R, (f) SERPINE1, (g) AURKB, (h) GNA13, (i) DLG2, (j) ACTN2. The disease-free survival information was based on GEPIA database, P < 0.05 was considered statistically different.

The present study analyzed the association between the top 20 hub gene expression and overall survival (OS), disease-free survival (DFS) of ccRCC patients by Kaplan-Meier analysis. The results shown that expression of RIPK4 (HR=0.58, log-rank *P* = 0.00042) was correlated with worse OS for ccRCC patients, as well as CDC42(HR=0.6, log-rank P=0.001), PTPN11 (HR=0.6, log-rank P=0.0011), KITLG (HR=0.55, log-rank P=0.00012), IGF1R (HR=0.4, log-rank P = 2.3e-08), FLT1 (HR=0.51, log-rank P=1.9e-05), AURKB (HR=2.1, log-rank P=2.8e-06), GNA13 (HR=0.63, log-rank P=0.0027), DLG2 (HR=0.63, log-rank P=0.0033) (Figure 3a-i). The expression of RIPK4 was correlated with worse DFS for ccRCC patients, as well as CDC42 (HR=0.68, log-rank P=0.033), PTPN11 (HR=0.65, log-rank P=0.021), KITLG (HR=0.56, log-rank P=0.002), IGF1R (HR=0.48, log-rank P=7.8e-05), SERPINE (HR=1.7, log-rank P=0.0065), AURKB (HR=2, log-rank P=0.00029), GNA13 (HR=0.68, log-rank P=0.04), DLG2 (HR=0.48, log-rank P=7.9e-05), ACTN2 (HR=2.2, log-rank P=2.3e-05) (Figure 4a-j).

### The Receiver Operating Characteristic (ROC) curve analysis of Hub genes

**Figure 5:**
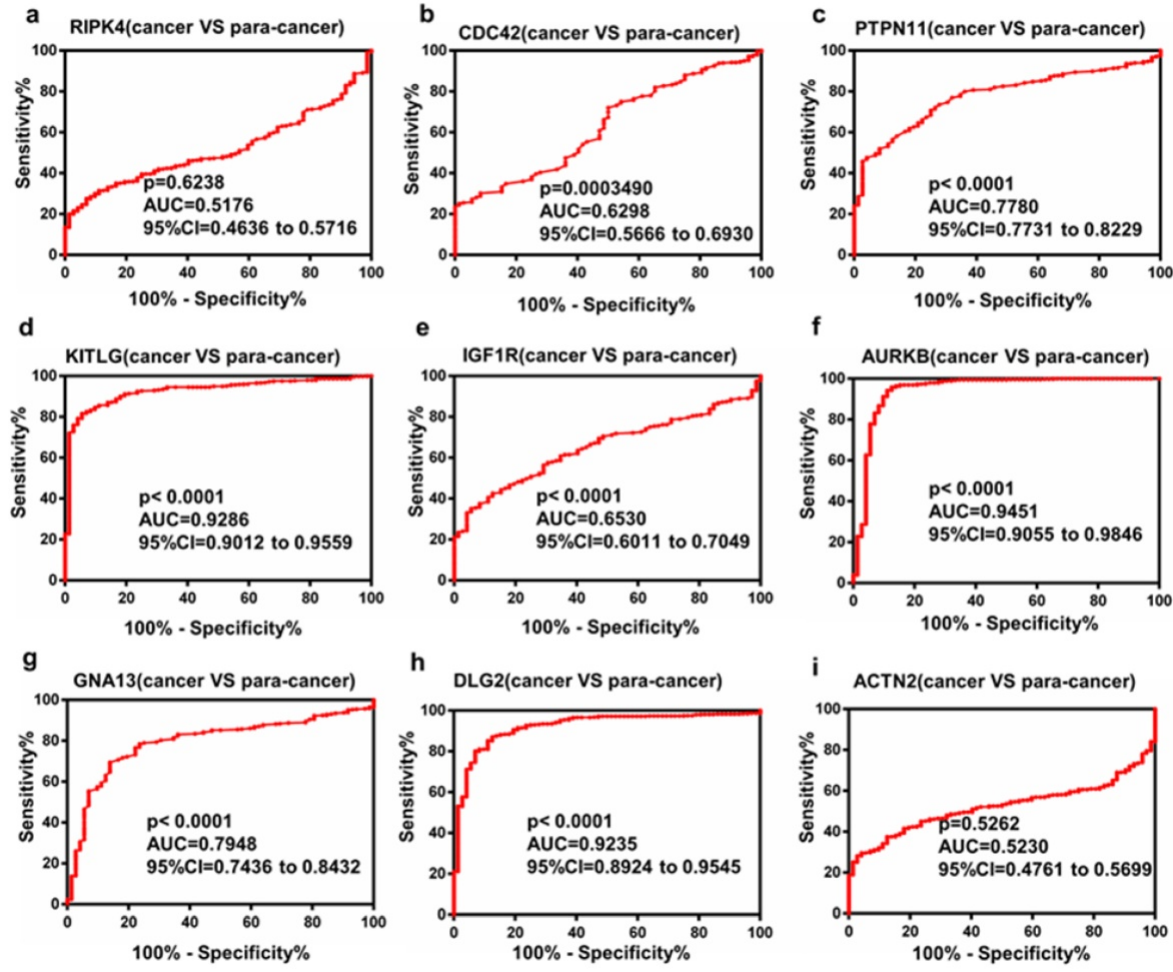
Diagnostic value of the selected top hub genes.

To investigate the diagnostic value of hub genes that may be potential prognostic biomarkers in ccRCC, ROC curves were evaluated. As shown in Figure. 5a-i, several genes could adequately distinguish ccRCC from paired normal tissues with an area under the curve (AUC) of 0.9235-0.9451. The results indicated that these hub genes may be effective diagnostic biomarkers for patients with ccRCC.

### The Expression of Hub Genes

**Figure 6:**
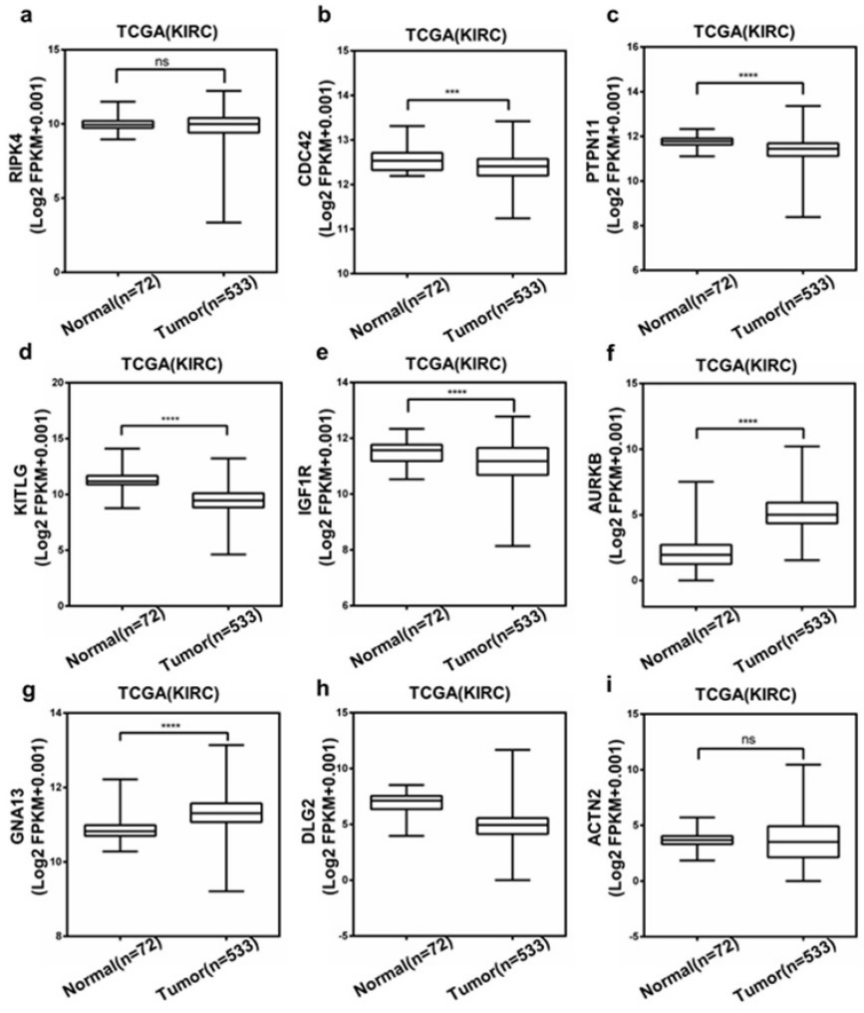
Expression of the selected top hub genes based on TCGA database.

Then, we chose RIPK4, CDC42, PTPN11, KITLG, IGF1R, AURKB, GNA13, DLG2 and ACTN2, which was associated with both OS and DFS, to evaluate the expression level between ccRCC and normal tissue using TCGA database. And as shown in Figure. 6a-i, compared to normal tissue, most of them have significant changes in ccRCC tissue. Only GNA13 and AURKB were elevated in ccRCC patient samples. Based on higher expression fold changes both in GSE47352 and TCGA databases and better diagnostic value, we selected AURKB to operate the further assessment.

### AURKB expression was correlated with various clinicopathological parameters in ccRCC

**Figure 7:**
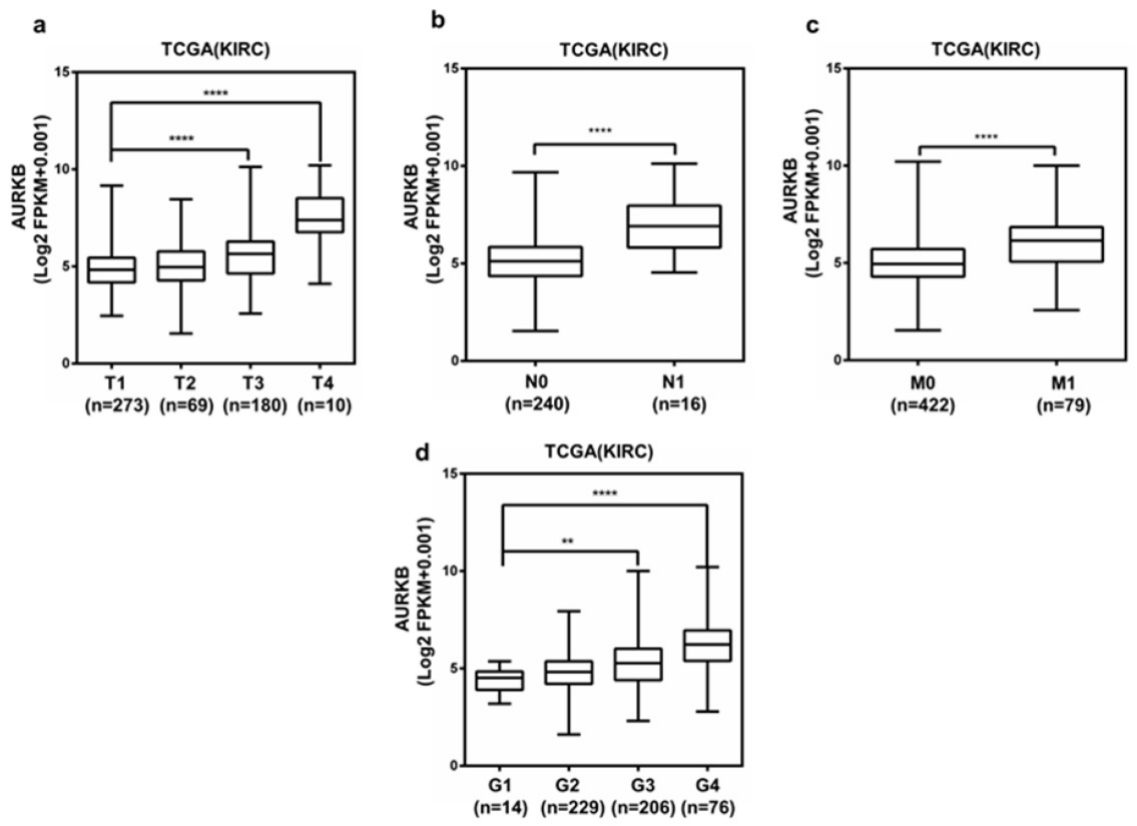
AURKB is overexpression in ccRCC, and is correlated with various clinicopathological parameters. The high mRNA expression of AURKB was correlated with various clinicopathological parameters: (a) T stage, (b) lymph node metastasis, (c) distant metastases, (d) G stage.

Analysis of the 533 ccRCC cases in the TCGA database shown that the upregulation of AURKB expression was significantly associated with higher pathological T stage, lymph node metastasis, distant metastasis and grade stage in ccRCC (Figure 7a-d). The expression of AURKB tended to elevate with enhancing tumor T stage and G grade. Clinicopathological information for the 526 ccRCC tissues in TCGA database was shown in Table 5. There was a significant correlation between high AURKB expression and these clinicopathological parameters, which was identical with the aforementioned results. These results indicated that AURKB could promote the progression and metastasis of ccRCC. Patients with high AURKB expression exhibited worse OS and DFS (Figure 3c, Figure 4d). To further explore prognostic value of AURKB, the prognostic value of each clinicopathological parameter, containing AURKB expression status, was estimated for OS (Table 6). Univariate Cox proportion hazard ratio (HR) analysis suggested that age (HR, 1.786; P<0.001), T stage (HR, 3.103; P<0.001), N stage (HR, 3.846; P<0.001), M stage (HR, 4.292; P<0.001), G grade (HR, 2.616; P<0.001) and AURKB expression status (HR, 2.761; P<0.001) were correlated with OS. Furthermore, multivariate analysis revealed that age (HR, 1.591; P=0.004), T stage (HR, 1.502; P=0.030), N stage (HR, 2.145; P=0.017), M stage (HR, 2.466; P<0.001), G grade (HR, 1.531; P=0.023) and AURKB expression (HR, 1.935; P<0.001) could be regarded as independent prognostic indicators of OS.

### Gene Set Enrichment Analysis

**Figure 8:**
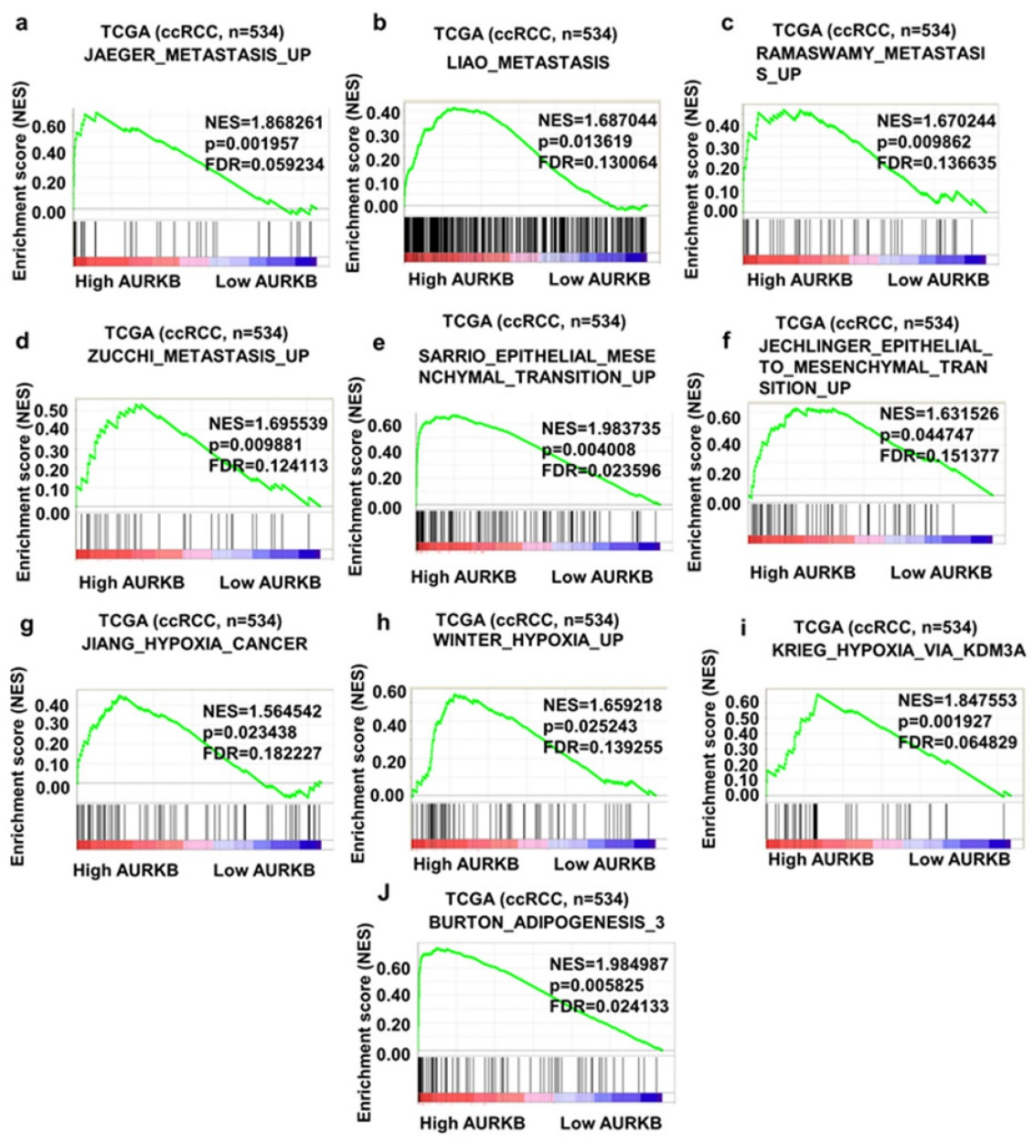
Gene set enrichment analysis (GSEA) of AURKB. 10 representative functional gene sets enriched in ccRCC with AURKB highly expressed were listed.

To obtain deeper insight into the function of the AURKB, GSEA was used to map into KEGG pathways and GO analysis database. 10 functional gene sets associated with metastasis or hypoxia pathway were shown, based on the cut-off criteria FDR < 0.05, and gene size ≥ 100, (Figure 8a-j). This result shown that high expression of AURKB was enriched in JAEGER_METASTASIS_UP gene set (NES=1.868261, p=0.001957, FDR=0.059234), LIAO_METASTASIS_UP gene set (NES=1.687044, p=0.013619, FDR=0.130064), RAMASWAMY_METASTASIS_UP (NES=1.670244, p=0.009862, FDR=0.136635) and ZUCCHI_METASTASIS_UP (NES=1.6955399, p=0.009881, FDR=0.124113). Other gene sets which were important pathway for ccRCC progression and metastasis were also associated with AURKB mRNA expression.

### AURKB expression and Biological function in ccRCC

**Figure 9:**
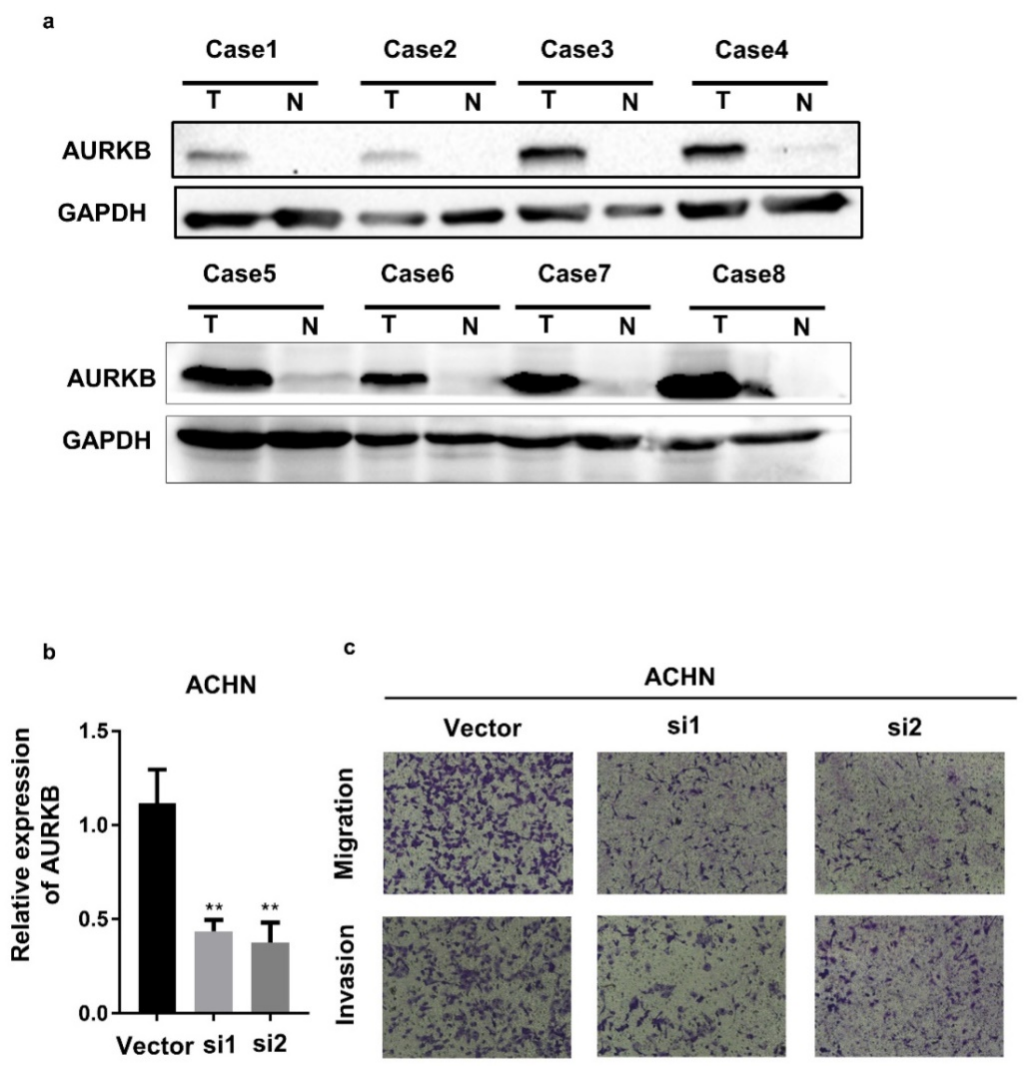
Expression and Biological function of AURKB. (a) The expression of AURKB in ccRCC tumor(T) and adjacent normal tissues(N), (b)AURKB knockdown in ACHN cell lines, (c) Transwell assay of AURKB in ACHN cell lines.

**AURKB** expression was examined in 8 pairs adjacent normal and ccRCC tumor tissues with western blot. We demonstrated that AURKB was overexpression in ccRCC tissues (Figure 9a). To investigate the mechanism of AURKB on the development of renal cancer, then we detected the biological function of AURKB. As the Figure 9c showed, AURKB knockdown significantly decreased the capacity of migration and invasion of ACHN cells.

## Discussion

ccRCC is the most common subtype of kidney cancer, and its prognosis is influenced by tumor progression correlated with complex gene interactions. Investigating the molecular markers of ccRCC is important for the survival of ccRCC patients. Despite significant efforts, the transformation of metastasis has thus far not been identified 18-20. Metastasis causes almost 90% of human cancer deaths 21.

In the current study, GEO2R was exploited to analyze the gene expression profile of GSE47352, including 4 metastatic and 5 non-metastatic ccRCC samples to explore the molecular mechanism of metastatic ccRCC and find some biomarkers, which might be useful therapeutic targets by using bioinformatics analysis. There was a total of 873 DEGs, including 503 up-regulated genes and 370 down-regulated genes, compared to the control ccRCC tissues. PPI network analysis and Cytoscape MCODE analysis were operated to evaluate protein-protein interactions and gene co-expression modules. Meanwhile, functional and pathway analysis were also operated to identify biological process and pathways of metastatic ccRCC patients.

According to the GO analysis of top 20 hub genes, biological process was mainly enriched in phosphatidylinositol-mediated signaling, positive regulation of DNA replication, regulation of phosphatidylinositol 3-kinase signaling, phosphatidylinositol phosphorylation, protein autophosphorylation. Molecular Function was mainly enriched in phosphatidylinositol-4,5-bisphosphate 3-kinase activity, protein tyrosine kinase activity, protein binding, protein kinase activity, Ras guanyl-nucleotide exchange factor activity. KEGG pathway analysis showed that top 20 hub genes were enriched in Ras signaling pathway, PI3K-Akt signaling pathway, Rap1 signaling pathway, HIF-1 signaling pathway. As we can see, most signaling pathways were correlated with Ras, phosphatidylinositol 3-kinase and protein tyrosine kinase pathway. Previous studies have reported that RAS families directly regulated various phosphatidylinositol 3-kinase isoforms 22. Meanwhile, one study investigated more than 400 ccRCC use different genemoic platform and found PI3K/AKT pathway was recurrently mutated, indicating this pathway was a potential therapeutic target 23. Phosphatidylinositol 3-kinase was demonstrated that metastatic RCC patients treated with anti-vascular inhibitory therapy who detected overexpression PI3K pathway have poor prognosis 24. Other studies also reported that PI3K signaling played a leading role in progression and metastasis of ccRCC 24-27. Not to mention protein tyrosine kinase pathway, NCCN, EAU have treated Tyrosine kinase inhibitor (TKI) drugs (solfatinib, sunitinib, etc.) as first-line treatment for metastatic renal cell carcinoma^28^, ^29^. Given their well-known pharmacology, therapeutic strategies to target them could demonstrate to be promising cancer treatment. Our study further confirmed the important regulatory role of signaling pathways such as RAS, PI3K pathway in renal cell metastasis. Therefore, monitoring of these signaling pathways may be helpful to further understanding the mechanism of metastasis and researching treatment.

Furthermore, PPI network analysis identified that RIPK4, TNF, CDC42, KNG1, PTPN11, KITLG, PTGS2, SYK, IGF1R, EPO, SERPINE1, FLT1, AURKB, GNA13, DLG2, ACTN2, CHEK1, FGF8, CD80 and MCHR2 had the highest degree of connectivity among DEGs. RIPK4 could promote cancer cell aggressiveness by upregulate VEGF-A 30. TNF mediates resistance to EGFR inhibition in cancer 31, 32. CDC42 as a member of Rho GTPases involved in cell proliferation and migration 33. The low expression of CDC42 was associated with poor prognosis and enhanced metastasis behavior 34-37. It was reported that PTPN11 play an important role in promoting progression and metastasis of cancer 38-41. Another hub gene, such as KITLG, PTGS2, SYK, FLT1 (VEGFR-1), GNA13, etc. could also regulate invasion and metastasis of cancer 42-48.

Cytoscape MCODE module analysis identified 3 modules with highly relevant expression pattern. Then GO biological progress analysis was operated to explore the signaling pathway of each module. These modules were mainly enriched in inflammatory response, G-protein coupled receptor signaling pathway, positive regulation of cytosolic calcium ion concentration. Inflammatory responses played leading roles at different stages of tumor progression, including initiation, malignant conversion, angiogenesis, invasion, and metastasis 49-51. G-protein coupled receptor was reported a central rule in tumor metastasis and induced angiogenesis 52.

To explore the diagnostic value of hub genes, ROC curves were used. The results revealed that mRNA level of several genes such as KITLG, AURKB, DLG2, could discriminate ccRCC tissues from normal tissues, yielding an AUC of 0.9235-0.9451. These data indicated that these hub genes may be potential biomarkers for the diagnosis of ccRCC.

AURKB, encoding a member of the aurora kinase subfamily of serine/threonine kinases, play an important role in tumorigenesis, inducing aneuploidy and genomic instability 53-57. In this study, we, for the first time, investigated the expression pattern, clinicopathological parameters and biological functions of AURKB in ccRCC. AURKB expression was uncovered to be upregulated in ccRCC tissues compared with in normal renal tissues, and was correlated with poor prognosis of patients with ccRCC. Multivariate regression analysis suggested that AURKB expression level was an independent prognostic factor for ccRCC; AURKB was a risk factor (HR=1.935; P<0.001). AURKB mRNA level could discriminate ccRCC tissues from normal tissues, yielding an AUC of 0.9451 (95% CI: 0.9055 to 0.9846; p < 0.0001). These results indicated that AURKB could be a potential and novel biomarker for diagnosis and prognosis of ccRCC patients. GSEA analysis was used to explore the biological function of AURKB by the expression of it in ccRCC TCGA database. The result showed that high expression of AURKB was associated with various metastasis gene data sets. Pervious study has reported that inhibition of Aurora kinases induced apoptosis and autophagy in leukemia cells via AURKB/p70S6K/RPL15 axis with the involvement of PI3K/Akt/mTOR, AMPK, and p38 MAPK signaling pathways 58. Other study uncovered that AURKB may activate the PTK2/PI3K/AKt/nuclear factor-KappaB pathway 59. These studies indicated that AURKB was involved in PI3K/AKt signal pathway. Epithelial-mesenchymal transition (EMT) plays a leading role in diversified biological and pathological processes, including metastasis and cancer cell drug resistance 60-62. EMT and Hypoxia pathway were both significantly correlated with the expression of AURKB. A hallmark of ccRCC is a clear cytoplasm that reflects increased lipid and glycogen deposition 63. Many studies reported that carcinogenesis of renal cancer could have very closely correlation with lipid metabolism 64, 65. In our study, the adipogenesis gene set was positively associated with the expression of AURKB. All these results indicated that AURKB may play an important role in invasion and metastasis of ccRCC.

In summary, by using a range of bioinformatics analyses, the present study has demonstrated the hub genes and important pathways that may be involved in ccRCC progression and metastasis, based on differentially expressed genes between metastatic and non-metastatic ccRCC samples. We also identified that some hub genes expression was associated with prognosis and diagnosis of ccRCC. Meanwhile, AURKB expression level was an independent prognostic factor for ccRCC. However, further explorations to confirm the function of the candidate biomarkers in ccRCC and to investigate the underlying molecular mechanisms of the genes involved in metastasis of ccRCC are needed.

## Figures and Tables

**Table 1:** Top 20 hub genes with higher degree of connectivity.

Gene	Degree of connectivity	Pvalue
RIPK4	52	2.11E-02
TNF	43	1.92E-03
CDC42	38	1.70E-02
KNG1	26	3.25E-02
PTPN11	23	3.27E-05
KITLG	22	3.61E-02
PTGS2	22	4.05E-02
SYK	22	3.12E-02
IGF1R	20	1.00E-02
EPO	20	4.50E-02
SERPINE1	17	2.64E-02
FLT1	17	6.29E-03
AURKB	17	6.59E-03
GNA13	16	1.57E-03
DLG2	16	8.08E-03
ACTN2	16	2.87E-02
CHEK1	16	4.55E-02
FGF8	16	7.36E-03
CD80	15	4.21E-02
MCHR2	15	7.91E-03

**Table 2:** Gene ontology analysis of top 20 hub genes associated with ccRCC Metastasis.

Category	Term	Count	%	P value
GOTERM_BP_DIRECT	GO:0048015~phosphatidylinositol-mediated signaling	5	25	5.40E-06
GOTERM_BP_DIRECT	GO:0045740~positive regulation of DNA replication	4	20	1.37E-05
GOTERM_BP_DIRECT	GO:0014066~regulation of phosphatidylinositol 3-kinase signaling	4	20	8.86E-05
GOTERM_BP_DIRECT	GO:0046854~phosphatidylinositol phosphorylation	4	20	1.54E-04
GOTERM_BP_DIRECT	GO:0046777~protein autophosphorylation	4	20	9.07E-04
GOTERM_CC_DIRECT	GO:0005886~plasma membrane	13	65	1.75E-04
GOTERM_CC_DIRECT	GO:0005615~extracellular space	8	40	2.71E-04
GOTERM_CC_DIRECT	GO:0031093~platelet alpha granule lumen	3	15	1.48E-03
GOTERM_CC_DIRECT	GO:0030175~filopodium	3	15	2.45E-03
GOTERM_CC_DIRECT	GO:0005576~extracellular region	7	35	4.66E-03
GOTERM_MF_DIRECT	GO:0046934~phosphatidylinositol-4,5-bisphosphate 3-kinase activity	4	20	4.38E-05
GOTERM_MF_DIRECT	GO:0004713~protein tyrosine kinase activity	4	20	4.22E-04
GOTERM_MF_DIRECT	GO:0005515~protein binding	18	90	6.64E-04
GOTERM_MF_DIRECT	GO:0004672~protein kinase activity	4	20	7.18E-03
GOTERM_MF_DIRECT	GO:0005088~Ras guanyl-nucleotide exchange factor activity	3	15	7.29E-03

**Table 3:** KEGG pathway analysis of top20 hub genes associated with Metastasis ccRCC.

Term	Count	%	P value
hsa04014:Ras signaling pathway	6	30	1.61E-04
hsa04151:PI3K-Akt signaling pathway	6	30	1.13E-03
hsa04015:Rap1 signaling pathway	5	25	1.44E-03
hsa04066:HIF-1 signaling pathway	4	20	1.63E-03
hsa05200:Pathways in cancer	6	30	2.03E-03
hsa05205:Proteoglycans in cancer	4	20	1.20E-02
hsa04520:Adherens junction	3	15	1.28E-02
hsa05203:Viral carcinogenesis	4	20	1.29E-02
hsa04510:Focal adhesion	4	20	1.30E-02
hsa04810:Regulation of actin cytoskeleton	4	20	1.39E-02

**Table 4:** GO biological progress analysis of Top three significant modules.

Module	Term	Count	P value	Genes
Module1	GO:0006954~inflammatory response	5	1.66E-05	KNG1, PTGER3, CXCL5, HRH4, CXCR3
	GO:0007186~G-protein coupled receptor signaling pathway	6	2.13E-05	TAS2R16, PTGER3, CXCL5, GRM7, TAS2R7, CXCR3
	GO:0007204~positive regulation of cytosolic calcium ion concentration	4	2.70E-05	KNG1, PTGER3, HRH4, CXCR3
	GO:0001580~detection of chemical stimulus involved in sensory perception of bitter taste	2	1.89E-02	TAS2R16, TAS2R7
	GO:0007200~phospholipase C-activating G-protein coupled receptor signaling pathway	2	3.10E-02	MCHR2, PTGER3
Module2	GO:0007204~positive regulation of cytosolic calcium ion concentration	3	1.89E-04	AGTR1, AVPR1A, TBXA2R
	GO:0045907~positive regulation of vasoconstriction	2	5.71E-03	AVPR1A, TBXA2R
	GO:0007186~G-protein coupled receptor signaling pathway	3	8.28E-03	AGTR1, AVPR1A, TBXA2R
	GO:0019722~calcium-mediated signaling	2	9.08E-03	AGTR1, AVPR1A

**Table 5:** Association between AURKB mRNA expression and clinicopathological parameters of patients with ccRCC.

		AURKB mRNA expression		
Parameter	Number	Low (n=263)	High (n=263)	P-value
Age (years)				
<60	243	122	121	
≥60	283	141	142	1.000
Gender				
Female	185	107	78	
Male	341	156	185	0.010
T stage				
T1 or T2	337	202	135	
T3 or T4	189	61	128	<0.001
N stage				
N0 or NX	510	261	249	
N1	16	2	14	0.004
M stage				
M0 or MX	448	244	204	
M1	78	19	59	<0.001
G grade				
G1 or G2	246	158	88	
G3 or G4	280	105	175	<0.001
TNM stage				
I + II	319	202	135	
III + IV	207	61	128	<0.001

**Table 6:** Univariate and multivariate analyses of AURKB mRNA expression and patient overall survival.

Risk factors	Univariate analysis			Multivariate analysis^c^		
Variable	HR^a^	95% CI^b^	P-value	HR	95% CI	P-value
Age (years)						
<60 (n=244)	1.786	1.304-2.445	<0.001	1.591	1.155-2.190	0.004
≥60 (n=282)						
Gender						
Female (n=183)	0.942	0.692-1.282	0.703			
Male (n=343)						
T stage						
T1 or T2 (n=355)	3.103	2.293-4.201	<0.001	1.502	1.041-2.167	0.030
T3 or T4 (n=191)						
N stage						
N0 or NX (n=510)	3.846	2.082-7.104	<0.001	2.145	1.144-4.023	0.017
N1 (n=16)						
M stage						
M0 or MX (n=447)	4.292	3.147-5.853	<0.001	2.466	1.719-3.540	<0.001
M1 (n=79)						
G grade						
G1 or G2 (n=246)	2.616	1.867-3.666	<0.001	1.531	1.061-2.210	0.023
G3 or G4 (n=280)						
AURKB						
Low (n=263)	2.761	1.987-3.836	<0.001	1.935	1.366-2.740	<0.001
High (n=263)						

^a^HR estimated from Cox proportional hazard regression model;^ b^CI of the estimated HR; ^c^Multivariate models were adjusted for T, N, M, G grade classification and age. CI, confidence interval; HR, hazard ratio.
